# Coal Fly Ash-Based Adsorbents for Tetracycline Removal: Comparative Insights into Modification and Zeolite Conversion

**DOI:** 10.3390/jox15020036

**Published:** 2025-03-01

**Authors:** Eric E. Houghton, Litha Yapi, Nils Haneklaus, Hendrik G. Brink, Shepherd M. Tichapondwa

**Affiliations:** 1 Water Utilization and Environmental Engineering Division, Department of Chemical Engineering, University of Pretoria, Pretoria 0002, South Africa; u18171941@tuks.co.za (E.E.H.); litha.yapi@up.ac.za (L.Y.); deon.brink@up.ac.za (H.G.B.); 2 Td Lab Sustainable Mineral Resources, University for Continuing Education Krems, 3500 Krems, Austria; nils.haneklaus@donau-uni.ac.at; 3 Unit for Energy and Technology Systems—Nuclear Engineering, North-West University, 11 Hoffman Street, Potchefstroom 2520, South Africa

**Keywords:** xenobiotics, tetracycline adsorption, fly ash modification, zeolite Na-P1, SDG 6, SDG 12

## Abstract

Emerging xenobiotics, such as tetracycline (TC), pose significant risks to both the environment and human health. Adsorption is a recognized method for removing these contaminants, and in this study, fly ash (FA), a by-product of coal combustion, was modified to develop adsorbents. Acid-modified FA (AM-FA) and base-modified FA (BM-FA) were prepared, and zeolite Na-P1 (ZNa-P1) was synthesized via hydrothermal treatment. Adsorption tests revealed that BM-FA and ZNa-P1 removed 76% and 90% of TC, respectively, compared to 35% with unmodified FA. AM-FA had the lowest performance, removing just 11% of TC. ZNa-P1’s superior performance was linked to its high zeolite purity, with a cation exchange capacity (CEC) of 6.37 meq/g and a surface area of 35.7 m^2^/g. Though BM-FA had a larger surface area of 110.8 m^2^/g, it exhibited a lower CEC of 3.42 meq/g. Adsorption efficiency was more closely related to CEC than surface area. Optimal TC removal with ZNa-P1 was achieved at a 7.5 g/L dosage and pH 5. The process followed pseudo second order kinetics and the Langmuir isotherm, with a maximum capacity of 46.34 mg/g at 30 °C. The adsorption thermodynamics indicated that the adsorption was endothermic and spontaneous. The adsorption mechanism of tetracycline on ZNa-P1 involved electrostatic attraction, hydrogen, and ion exchange. This study aligns with SDGs 6 (Clean Water and Sanitation) and 12 (Responsible Consumption and Production).

## 1. Introduction

Water pollution poses a significant threat to ecosystems and human health [1]. Emerging contaminants (ECs) are a class of xenobiotics that have garnered increasing attention due to their persistence in ecosystems and resistance to conventional biological wastewater treatment methods [2]. One prominent emerging contaminant is tetracycline (TC), one of the most widely used antibiotics [3]. TC is not fully metabolized by humans and animals, with approximately 50–80% excreted in its original form through urine and faeces [4]. Many wastewater treatment plants (WWTPs) use activated sludge processes that are not fully effective in removing TC, resulting in the discharge of TC into the environment [5]. The hydrophilic nature, low volatility, and extended half-life of TC enable it to persist in aquatic environments for extended periods [6,7]. One of the primary concerns associated with TC in the aquatic environment is the development of antibiotic-resistant bacteria (ARB), which pose a significant risk to human health [8]. Infections caused by ARB are associated with higher rates of mortality and morbidity [9]. Various methods are available for removing TC from aqueous solutions, including ozonation, adsorption, membrane filtration, and ion exchange [10]. Among these, adsorption is regarded as one of the simplest and most cost-effective treatment methods of TC removal. Although activated carbon is a widely used adsorbent, its high cost limits its broader application in wastewater treatment [11]. Several low-cost alternatives, including manufacturing by-products (e.g., fly ash, red mud), clay minerals, and biological materials (e.g., chitin, chitosan), offer promising solutions [12].

Fly ash (FA) is a solid waste primarily generated by coal-fired thermal power plants, with an estimated global production of 600 million tonnes annually [13]. However, only 30% of FA is reused [14]. Due to the presence of hazardous components, FA must be disposed of in controlled landfills, which is both costly and time-consuming [15]. As noted, FA can be repurposed as a low-cost adsorbent for removing pollutants from water [12]. However, its adsorption capacity is limited by its low surface area and crystalline structure. To address this, researchers have explored various modification techniques to enhance FA’s adsorption performance [16]. Given its high silica and alumina content, FA can serve as cheap precursor for synthesizing zeolites, which can serve as effective adsorbents [13]. The application of FA as an adsorbent is well-established, with over 4594 documents identified using the SCOPUS search engine and the keywords ‘Fly ash’ and ‘adsorption’. However, there is a notable gap in the utilization of FA for EC adsorption, with only 12 studies focusing on this application. Specifically, only three articles, by Bandura, Bialoszewska [17], Sun, Dai [18] and Ren, Wang [19], have explored the use of FA for TC adsorption. Notably, none of these studies compared the performance of unmodified FA, modified FA, and zeolites as adsorbents for TC adsorption. This gap underscores the novelty of the present study.

This study aimed to develop various adsorbents from fly ash through modification and hydrothermal synthesis. A preliminary adsorption experiment was conducted to evaluate the effectiveness of the derived adsorbents in removing tetracycline from aqueous solutions. The best-performing adsorbent was selected for further studies to determine the optimal dosage and pH for tetracycline adsorption, as well as to model the adsorption kinetics and isotherms of the process. By utilising waste FA for the purposes of removing pollutants from aqueous solutions, this study aligns with the SDGs 6 and 12 by improving wastewater treatment and sustainable utilization of waste products.

## 2. Materials and Methods

### 2.1. Materials

Fly ash (FA) was collected from the Matla Power station located in Kriel, Nkangala, Mpumalanga, South Africa (S26°16′53.84″, E29°8′18.25″). Tetracycline (TC) was purchased from Sigma-Aldrich, Steinheim, Germany. Hydrochloric acid (HCl~32%), sodium hydroxide (NaOH) pellets, acetic acid (99%), and potassium dihydrogen phosphate were purchased from Glassworld, Johannesburg, South Africa. Potassium hydrogen phthalate was purchased from Hopkins and Williams Ltd., London, UK. Sodium acetate trihydrate was purchased form SAARCHEM, Germiston, South Africa. Deionized (DI) water used in this study was from an Elga Purelab Chorus unit. Filter paper used was a qualitative grade Whatman no. 40 with a pore size of 8 µm. Potassium hydrogen phthalate (pH 2–5), potassium dihydrogen phosphate (pH 6–8), and sodium tetraborate (pH 9) buffers were used to regulate the pH of solutions.

### 2.2. Preparation of Acid-Modified Fly Ash (AM-FA)

The acid modification synthesis was adopted from Eteba, Bassyouni [20]. A 10 g quantity of FA was mixed with 50 mL of HCl solution (30 w/w%). The resulting slurry was poured into a glass sealable flask and placed in an oven at 100 °C for 24 h. After 24 h, the slurry was filtered using vacuum filtration. The resulting filter cake was washed with DI water several times. After which, it was dried in an oven for 24 h at 60 °C.

### 2.3. Preparation of Base-Modified Fly Ash (BM-FA)

The base modification was adopted from Wulandari, Saefumillah [21]. A 20 g quantity of FA was mixed with 160 mL of 3.5 M NaOH solution. The resulting slurry was poured into a Teflon-lined autoclave and placed in an oven at 100 °C for 24 h. After 24 h, the slurry was filtered using vacuum filtration. The resulting filter cake was washed with DI water several times till the filtrate wash-water had a pH lower than 10. After which, it was dried in an oven for 24 h at 60 °C.

### 2.4. Preparation of Zeolite Na-P1 (ZNa-P1)

**Figure 1:**
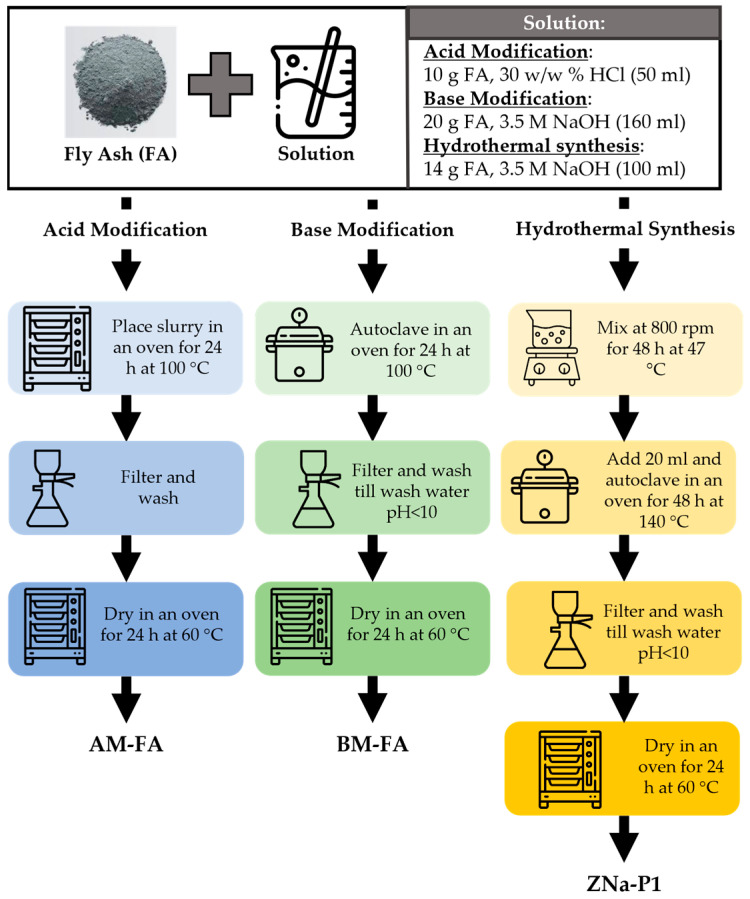
Schematic representation of experiments.

The zeolite synthesis was adopted from Musyoka, Petrik [22] and consisted of two steps: ageing and hydrothermal treatment. In the ageing process, 14 g of FA was mixed with 100 mL of 3.5 M NaOH solution. The mass ratio of FA:NaOH was 1:1. The resulting slurry was poured into a sealable polyethylene bottle. The bottle was placed into a silicone-oil bath on a magnetic stirrer and stirred at 800 rpm and 47 °C for 48 h. The hydrothermal treatment step involved the addition of approx. 20 mL of DI water to the aged-slurry, after which it was mixed to obtain homogeneity. The slurry was transferred to a Teflon-lined autoclave and heated in an oven at 140 °C for 48 h. Following the hydrothermal treatment, the resulting zeolite mixture was filtered using vacuum filtration. The zeolites were then washed multiple times until the pH of the filtrate wash water was below 10. After which, it was dried in an oven for 24 h at 60 °C. Table 1 presents a summary of the conditions for the material synthesis, and Figure 1 gives a schematic representation of the experiments.

### 2.5. Adsorption Experiments

Firstly, a 200 ppm TC stock solution was prepared. The batch adsorption studies were carried out in 30 mL glass vials, and the experimental solutions were prepared by diluting the TC stock with a pH buffer. Fly ash and the derived adsorbents had a strong alkalinity, which influenced the pH of the solutions. The pH buffers were used to regulate the pH and avoid pH fluctuations. Kao, Tzeng [23] noted that pH fluctuations near the pKa of an adsorbate might impact adsorption more than the presence of a pH buffer. Table 2 summarizes the adsorption experiments conducted.

All experimental samples were agitated on an orbital shaker at a rotation speed of 250 rpm. After adsorption, a 2 mL sample was centrifuged at 10,000 rpm for 1 min to separate the adsorbent from solution, and the TC concertation was determined from the supernatant. The initial and final TC concentrations were determined using a VWR UV-1600PC spectrophotometer at an interval of 1 nm from 500 nm to 300 nm. The sample was diluted by a factor of 2 with 0.1 M NaOH. At a high pH, tetracycline produces a more intense yellow colour that is more easily detectable on the UV-VIS Spectrophotometer. The calibration curve was developed, with the observed peak wavelength at 381 nm. The percentage removal and adsorption capacity at time t and the adsorption capacity at equilibrium $q_{e}$ were determined using Equations (1), (2), and (3), respectively.

(1) $$\mathrm{removal}\;\left(\%\right)=\frac{C_{O}-C_{e}}{C_{O}}\times 100$$

(2) $$q_{t}=\frac{(C_{O}-C_{t})V\;}{m}$$

(3) $$q_{e}=\frac{\left(C_{O}-C_{e}\right)V}{m}$$

where $C_{O}$, $C_{t}$, and $C_{e}$ (ppm) are the initial concentration, concentration at time t, and equilibrium concentration, respectively, $q_{e}$ is the adsorption capacity at equilibrium (mg/g), $q_{t}$ is the adsorption capacity at time t, V is the volume of the solution (L), and m is the mass of adsorbent used (g).

### 2.6. Adsorbent Characterization

XRD analysis was conducted with a PANalytical X’Pert Pro powder diffractometer in θ-θ configuration with an X’Celerator detector and variable divergence and fixed receiving slits with Fe filtered Co-Kα radiation (λ = 1.789 Å). The mineralogy was determined by selecting the best-fitting pattern from the ICSD database to the measured diffraction pattern using X’Pert Highscore plus software (version 5.1). The crystallinity of zeolite Na-P1 was determined using Equation (4) [24]:

(4) $$\mathrm{Crystallinity}\;\left(\%\right)=\frac{\sum \;\mathrm{Area}\;\mathrm{of}\;\mathrm{zeolite}\;\mathrm{peaks}}{\sum \;\mathrm{Area}\;\mathrm{of}\;\mathrm{all}\;\mathrm{peaks}\;\mathrm{in}\;\mathrm{scan}\;}\times 100$$

XRF analysis was conducted using a Thermo Fisher ARL Perform’X Sequential XRF instrument with Uniquant software (version 5.0). The software analysed all elements in the periodic table between Na and U, but only elements found above the detection limits were reported. BET surface area was measured on a Micromeritics TriStar II, with a liquid nitrogen temperature of 77.35 K. Degassing for BET Analysis was performed at 110 °C for 18 h before analysis. The morphology of the synthesized particles was captured on a Zeiss Crossbeam 540 FEG SEM instrument using the Oxford instruments detector and Aztec 3.0 software SP1. Strips of carbon tape, each with the relevant sample, were attached to an aluminium plate before being coated with carbon. The carbon coater used is an SEM auto-coating unit E2500 (Polaron Equipment Ltd., Hertfordshire, UK). FTIR was conducted on some of the adsorbents before and after adsorption on a Shimadzu IRSpirit-TX Fourier transform infrared spectrophotometer equipped with a quartz attenuated total reflectance accessory (QATR). All FTIR scans were recorded at a resolution of 2 cm^−1^ for 45 scans from 4700 cm^−1^ to 350 cm^−1^. Note that the scans represent an average of the 45 scans. The zeta potential (ζ-potential) was characterized using dynamic light scattering (DLS) on a Zetasizer Nano-ZS instrument (Malvern Instruments, Malvern, UK). Samples were prepared from a stock solution of 100 ppm of ZNa-P1 and diluted to 1 ppm. The 1 ppm solutions were then adjusted to various pHs using 0.1 M NaOH and 0.1 M HCl. The samples were left for 24 h before zeta potential analysis.

### 2.7. Cation Exchange Capacity (CEC) Measurement for the Adsorbents

The CEC of FA and AM-FA was determined using a method adopted from Woolard and van der Horst [25]. One gram of the ash sample was shaken for 15 min with 33 mL of 1 M sodium acetate then centrifuged for 15 min. The extracted ash sample was again shaken in a 1 M sodium acetate solution for 15 min. This procedure was carried out a total of three times. The sample was then washed four times in ultra-pure water. Finally, the sample was shaken for 15 min with 33 mL of 1 M ammonium acetate solution then centrifuged for 15 min. The supernatant was kept aside for analysis, and the extracted sample was again shaken in 1 M ammonium acetate solution for 15 min. This was carried out a total of three times. The final accumulative volume of supernatants was approximately 100 mL. The concentration of sodium in the final solution was determined using atomic adsorption spectroscopy with a PerkinElmer AAnalyst 400.

The CEC of ZNa-P1 and BM-FA was determined using a method adopted from Musyoka, Petrik [26]. A total of 0.5 g of zeolite was shaken with 25 mL of 1 M ammonium acetate for 15 min, followed by centrifugation for 15 min. The supernatant was collected, and the zeolites were shaken again with 1 M ammonium acetate. This process was repeated four times in total, resulting in a cumulative supernatant volume of approximately 100 mL. The concentration of sodium ions in the final solution was determined using atomic absorption spectroscopy with a PerkinElmer AAnalyst 400.

## 3. Results

### 3.1. XRD Analysis

**Figure 2:**
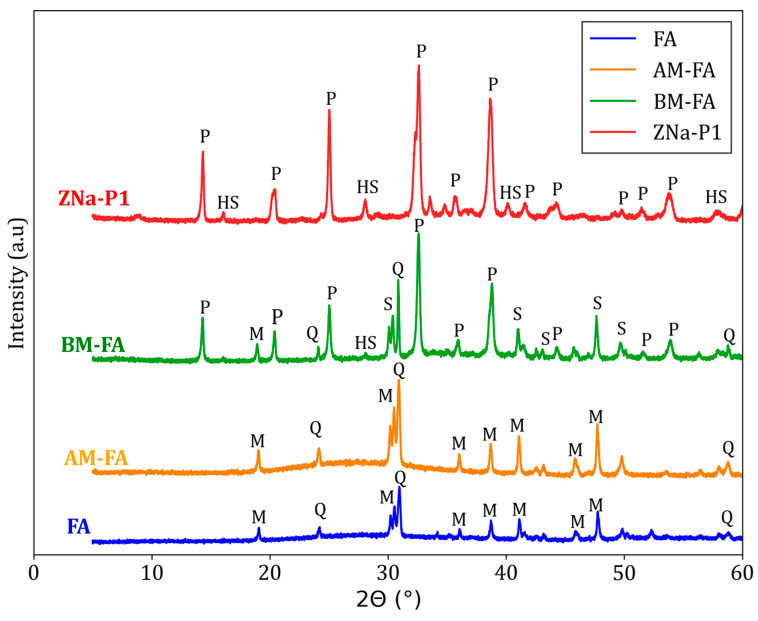
XRD Patterns for FA, AM-FA, BM-FA, and ZNa-P1. The phases indicated in the XRD patterns are as follows: mullite (M), quartz (Q), sillimanite (S), hydroxy-sodalite (HS), and zeolite Na-P1 (P).

XRD analysis was conducted to evaluate the change in crystallinity of the FA after modification. The XRD patterns for FA, AM-FA, BM-FA, and ZNa-P1 are shown in Figure 2. The XRD pattern for FA indicated that the major crystalline phases were mullite and quartz.

There was no significant change noted in the XRD pattern of AM-FA after acid modification. Taufiq, Hidayat [27] and Wulandari, Saefumillah [21] also noted that there was no substantial change after acid modification beyond the reduction of the amorphous phase. The XRD pattern of BM-FA showed the presence of zeolite Na-P1. However, the occurrence of mullite and quartz peaks indicates only partial digestion of these phases for zeolitization [28]. The XRD pattern of ZNa-P1 showed two main phases being zeolite Na-P1 and hydroxy-sodalite. Hydroxy-sodalite is another zeolite phase, which is considered an impurity in the zeolite Na-P1 synthesis [26]. The mullite and quartz peaks completely disappeared, indicating full digestion of these phases for zeolitization, and suggesting a high conversion of the FA to a zeolite Na-P1 [28]. The percentage crystallinity of zeolite Na-P1, as calculated using Equation (4), in BM-FA and ZNa-P1 was 43% and 86%, respectively.

### 3.2. XRF Analysis

The chemical composition of FA, AM-FA, BM-FA, and ZNa-P1 was analysed using XRF and is shown in Table 3. The fly ash was classified as Class F as per the ASTM C618 standard, since the combined SiO_2_, Al_2_O_3_, Fe_2_O_3_ content exceeded 70% and there was a low CaO content (<18%) [29,30]. The results indicate a significant decrease in metal cations, i.e., aluminium, iron, magnesium, and calcium, after acid modification. The observed increase in silicon was attributed to a reduction of other elements and the fact that silicon is more resistant to acidic attack [21,31]. After base modification, there was a drop in silicon and aluminium content and an increase in the sodium content. The NaOH could have leached out the silica and aluminium into the solution. The large sodium increase also indicates that zeolitization took place during the base modification [29]. The ZNa-P1 has a composition similar to BM-FA. Notably, the ZNa-P1 has a higher sodium composition than the BM-FA, indicating more zeolitization occurred in the production of ZNa-P1.

### 3.3. Morphology and Surface Area Analysis

**Figure 3:**
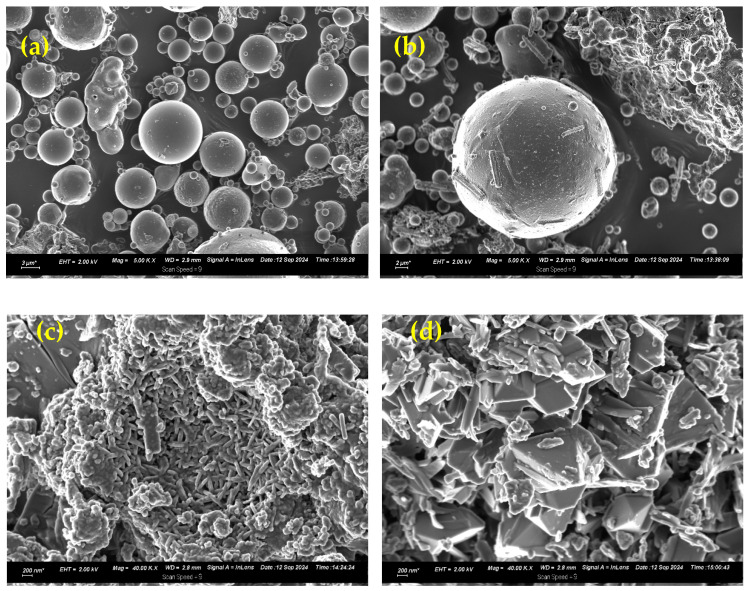
SEM images for (**a**) FA, (**b**) AM-FA, (**c**) BM-FA, and (**d**) ZNa-P1. Note that due to the differences in particle sizes, the magnification for FA and AM-FA is 5000 and for BM-FA and ZNa-P1 is 40,000.

Figure 3 shows the SEM images for FA, AM-FA, BM-FA, and ZNa-P1. The surface morphology of FA consists of spherical and smooth particles. No significant change in the surface morphology was observed after acid modification to AM-FA. The surface morphology of BM-FA showed a substantial change after base modification. The particles transformed from smooth spherical shapes to more irregular-shaped crystalline particles. The surface morphology of ZNa-P1 showed more needle-like crystalline shapes, which is the typical morphology of zeolite Na-P1 [28]. Table 4 gives the specific surface area for FA, AM-FA, BM-FA, and ZNa-P1 determined using the BET method. The acid modification increased the specific surface area by approximately 100%. The base modification showed the largest increase in specific surface area, approximately a ten-fold increase. The ZNa-P1 adsorbent had a surface area three times as large as FA. After alkali activation, the particle size of FA decreases due to alkali etching of the surface of the FA particles, resulting in an increase in surface area [32]. The large difference in particle sizes between FA and BM-FA/ZNa-P1 is seen in Figure 3.

### 3.4. Cation Exchange Capacity (CEC) Analysis

**Figure 4:**
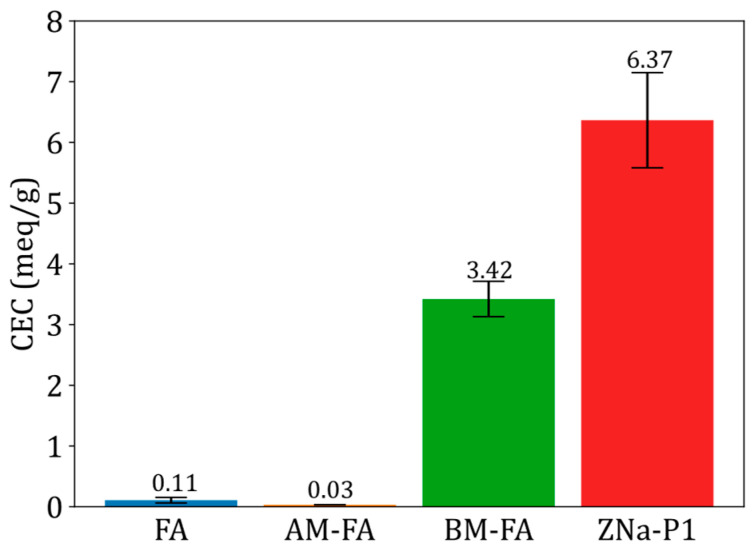
Cation exchange capacity for FA, AM-FA, BM-FA and ZNa-P1.

Figure 4 shows the cation exchange capacity for FA, AM-FA, BM-FA, and ZNa-P1. Both FA and AM-FA had significantly lower CEC values as compared to BM-FA and ZNa-P1. The higher CEC values for BM-FA and ZNa-P1 were attributed to the presence of zeolite Na-P1 in these materials [33]. As previously stated, the percentage of crystallinity of BM-FA and ZNa-P1 was 43% and 86%, respectively. Note that the ZNa-P1 had both a higher zeolite crystallinity and higher CEC value, suggesting a correlation between cation exchange capacity and zeolite crystallinity. Zheng, Ma [34] found a similar conclusion, that the CEC values were directly proportional to the zeolite crystallinity.

### 3.5. Comparison of Adsorbents for Tetracycline Adsorption

**Figure 5:**
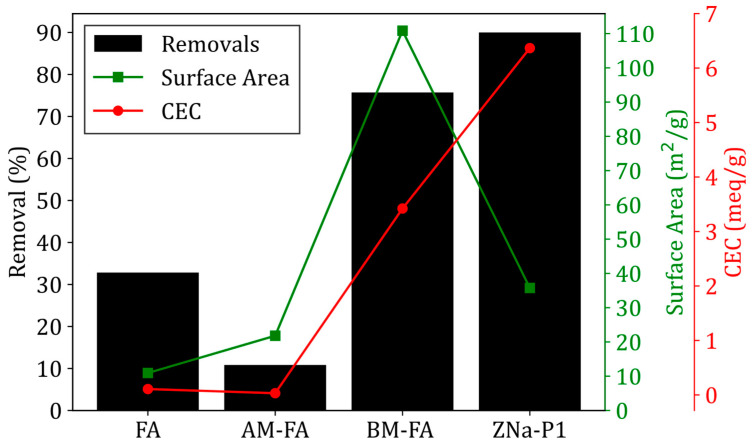
The percentage of removal, surface area (**right-axis**), and CEC (far-**right-axis**) of FA, AM-FA, BM-FA, and ZNa-P1 (conditions for removal experiments: pH = 5, dosage = 5 g/L, contact time = 2 h, initial concentration = 40 ppm).

The preliminary adsorption test was conducted under the same set of conditions for FA, AM-FA, BM-FA, and ZNa-P1. The percentages of removal of TC for FA, AM-FA, BM-FA, and ZNa-P1 are shown in Figure 5. The percentage of removal of FA was 35%; after acid modification, the percentage of removal was reduced to 11%. Despite an increase in the surface of AM-FA, it exhibited a lower CEC value.

In contrast, BM-FA and ZNa-P1 demonstrated significantly higher TC removal efficiencies, at 76% and 90%, respectively. This improvement was attributed to their high surface areas and CEC values. It should be noted that ZNa-P1 had a lower surface area than BM-FA but a higher CEC value. The higher percentage of removal of ZNa-P1 could be attributed to the high CEC value due to the purer zeolite phase (Na-P1) present in ZNa-P1. The adsorption of TC is strongly correlated to the CEC of a material and not the surface area. CEC refers to the ability of a material to adsorb exchangeable cations. A higher CEC signifies that the material can more effectively adsorb cations through electrostatic forces [35].

Given its high TC removal efficiency, ZNa-P1 was selected as the sole adsorbent for further adsorption experiments to optimize time and resources.

### 3.6. The Effect of Adsorbent Dosage

**Figure 6:**
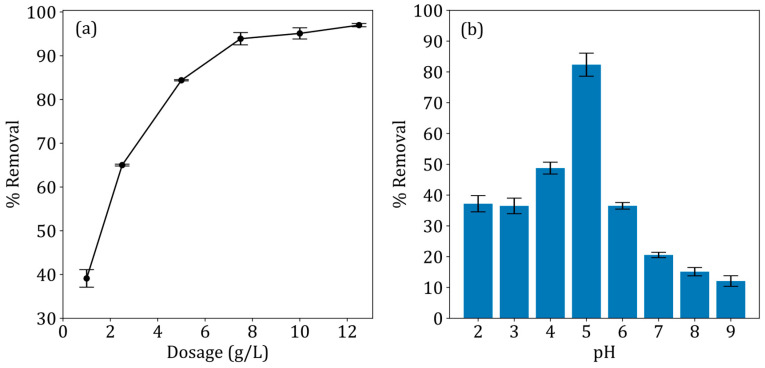
(**a**) The effect of adsorbent dosage and (**b**) the effect of pH on tetracycline removal using ZNa-P1.

The effect of adsorbent dosage on the removal of tetracycline is shown in Figure 6a. The results indicate that an increase in the adsorbent dosage increased the removal of tetracycline. This could be attributed to the increase in active sites for adsorption at higher dosages. The optimal dosage was 7.5 g/L, as there was no substantial increase in the percentage removal of tetracycline after a dosage of 7.5 g/L.

### 3.7. The Effect of pH

**Figure 7:**
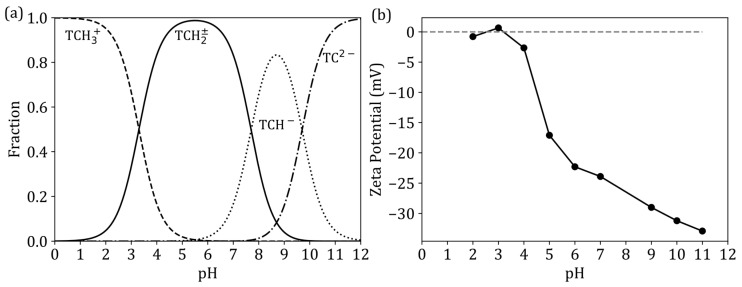
(**a**) The speciation of tetracycline as a function of pH [37] and (**b**) Zeta potential of ZNa-P1 as a function of pH, determined using a ZetaSizer.

The effect of pH on the removal of tetracycline is shown in Figure 6b. Due to the amphoteric nature of tetracycline, it can exist as a cationic, anionic, or neutral species, based on the pH [36]. Figure 7a presents TC-speciation as a function of pH, and Figure 7b presents the zeta potential of ZNa-P1 as a function of pH determined using a ZetaSizer. The highest percentage of removal was noted at a pH of 5; therefore, pH 5 was determined to be the optimal pH. At a pH of 5, tetracycline is neutral, and the ZNa-P1 surface is negatively charged. According to Jia, Zhou [37], though tetracycline may not have a net charge (neutral), its adsorption behaves as though it was positively charged. This suggest there is an electrostatic attraction at a pH of 5. At a pH < 5, tetracycline adsorption decreases. At a pH < 5, tetracycline becomes cationic, whereas the ZNa-P1 surface becomes neutral, and thus the electrostatic attraction between TC and ZNa-P1 no longer exists. At pH > 5, tetracycline adsorption decreases. At pH > 5, tetracycline becomes anionic, and the ZNa-P1 surface possesses a negative charge, causing electrostatic repulsion between ZNa-P1 and TC, reducing adsorption. Note that as the pH increases, the negative charge on the surface of ZNa-P1 increases, increasing electrostatic repulsion, and TC adsorption decreases.

### 3.8. Adsorption Kinetics

**Figure 8:**
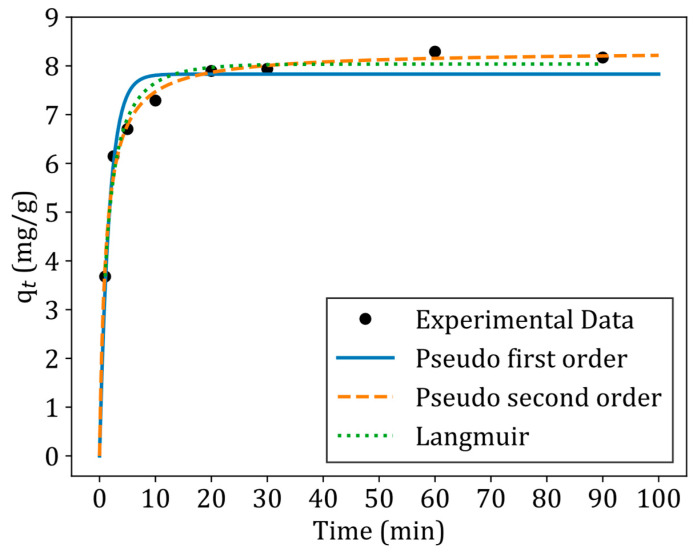
Adsorption kinetics of tetracycline on ZNa-P1 with the fitted adsorption kinetic models (conditions for kinetic experiments: pH = 5, Dosage = 7.5 g/L, initial concentration = 60 ppm).

The adsorption kinetics were modelled using non-linear regression. The models used to describe the adsorption kinetics were the pseudo first order model (PFO), pseudo second order model (PSO), and the Langmuir kinetic model, given in Equations (5), (6), and (7), respectively [38]:

(5) $$q_{t}=q_{e}\left(1-e^{-k_{1}t}\right)$$

(6) $$q_{t}=\frac{k_{2}q_{e}^{2}t}{1+k_{2}q_{e}t}$$

(7) $$\frac{dq_{t}}{dt}=k_{a}C_{t}\left(1-\frac{q_{t}}{q_{max}}\right)-k_{d}\frac{q_{t}}{q_{max}}$$

where $k_{1}$ (1/min) and $k_{2}$ (g/(mg·min) are the rate constants for the PFO and PSO, $q_{max}$ (mg/g) is the maximum adsorption capacity constant, $k_{a}$ (L/g/min), $k_{d}$ (mg/L/min) are the adsorption and desorption rate constants for the Langmuir model. Figure 8 shows the adsorption kinetics of tetracycline with the fitted pseudo first order, pseudo second order and Langmuir kinetic models. Table 5 presents a summary of model parameters and statistical parameters for each fitted model.

The pseudo second order model exhibited the lowest squared error and highest R^2^ value, indicating that the adsorption kinetics of tetracycline were best described by this model. According to Molina-Calderón, Basualto-Flores [39], the pseudo second order model is useful for explaining adsorption where the rate-controlling steps involve chemisorption mechanisms (such as complexation) or ion exchange. However, Bandura et al. [17] caution that the adsorption mechanism cannot be definitively determined by the PFO or PSO models. The best fit of the PSO model in this case suggests that chemisorption or ion exchange could be the dominant mechanism for tetracycline adsorption onto ZNa-P1.

### 3.9. Adsorption Isotherms

**Figure 9:**
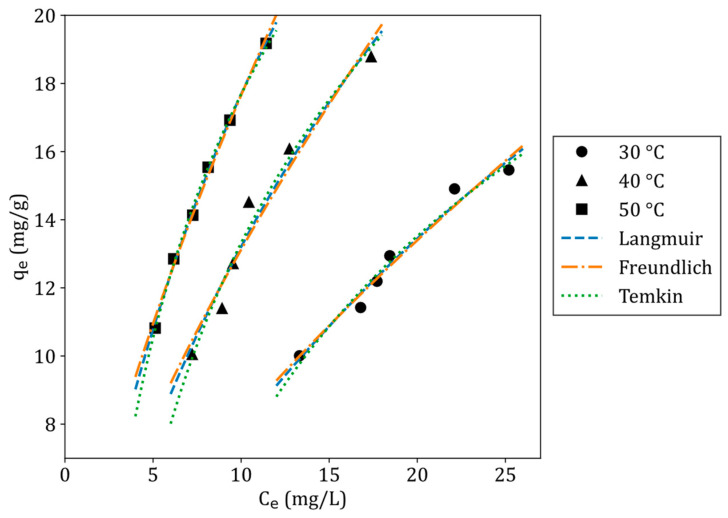
Adsorption isotherms of tetracycline on ZNa-P1 at 30 °C, 40 °C, and 50 °C fitted with the adsorption isotherm models (conditions for isotherm experiments: pH = 5, dosage = 7.5 g/L, contact time = 4 h).

The adsorption isotherms were modelled using non-linear regression. The models used to describe the adsorption isotherms were the Langmuir isotherm model, Freundlich isotherm model, and the Temkin isotherm model, given in Equations (8), (9), and (10), respectively [40]:

(8) $$q_{e}=\frac{q_{m}K_{L}C_{e}}{1+K_{L}C_{e}}$$

(9) $$q_{e}=K_{f}C_{e}^{\frac{1}{n}}$$

(10) $$q_{e}=B_{T}\ln(A_{T}C_{e})\;\;$$

where $q_{m}$ (mg/g) is the maximum adsorption capacity, $K_{L}$ (L/mg) is the Langmuir constant, $K_{f}$ is the Freundlich isotherm constant (mg/g), n is the adsorption intensity, $B_{T}\;$(J/mol) is the Temkin constant related to heat of sorption, and $A_{T}$ (L/mg) is the Temkin equilibrium binding constant. Figure 9 shows the adsorption isotherms of tetracycline at 30 °C, 40 °C, and 50 °C fitted with Langmuir, Freundlich, and Temkin isotherm models. Table 6 presents a summary of the adsorption isotherm model parameters and statistical parameters for each isotherm model. The Langmuir isotherm model showed the lowest squared error and highest R^2^ value at all temperatures, except for 40 °C, indicating that the adsorption isotherms of tetracycline were best described by this model. The Langmuir model’s best fit suggests that tetracycline adsorption onto ZNa-P1 occurred as a monolayer on a homogeneous surface [41]. Although the R^2^ values were similar across the models, the Langmuir isotherm provides a more theoretically sound representation of tetracycline adsorption onto ZNa-P1. Unlike the Freundlich and Temkin models, which are empirical or semi-empirical, the Langmuir model is grounded in the assumption of monolayer adsorption on a homogeneous surface. It also provides valuable parameters, such as the maximum adsorption capacity (q_max_), which are essential for designing and scaling up adsorption systems [42,43,44]. Table 7 presents the maximum adsorption capacities ($q_{m}$) of various adsorbents for tetracycline removal. Notably, the β-cyclodextrin-modified zeolite Na-P1 exhibited a lower adsorption capacity, implying that the organic modification did not significantly enhance tetracycline adsorption.

### 3.10. Adsorption Thermodynamics

The Gibbs free energy (ΔG°) can be calculated according to Equation (11). The entropy change (ΔS°) and enthalpy change (ΔH°) can be calculated using Equation (12).

(11) $$\Delta G{}^{\circ}=-RT\;\ln\left(K_{eq}\right)\;$$

(12) $$\ln\left(K_{eq}\right)=\frac{\Delta S{}^{\circ}}{R}-\frac{\Delta H{}^{\circ}}{RT}$$

where T (K) is the absolute temperature, R is the universal gas constant (8.314 J/mol/K), and $K_{eq}$ is the standard thermodynamic equilibrium constant of adsorption [51]. The dimensionless constant $K_{eq}$ can be determined from the Langmuir constant $K_{L}$ using Equation (13),

(13) $$K_{eq}=K_{L}\times 1000\times M_{w}\times \frac{C{{}^{\circ}}_{Absorbate}}{\gamma_{Absorbate}}$$

where $M_{w}$ (g/mol) is the molecular weight of the absorbate, $C{{}^{\circ}}_{absorbate}$ (mol/L) is the standard concentration of the adsorbate, and $\gamma_{absorbate}$ is the activity coefficient of the adsorbate [52]. Similar to Tran, Lima [52], both $C{{}^{\circ}}_{absorbate}$ and $\gamma_{absorbate}$ will be assumed to be in unity. Table 8 presents a summary of the thermodynamic parameters for the adsorption of tetracycline onto ZNa-P1. The negative value of ΔG° signifies that the adsorption process occurs spontaneously. The positive ΔS° indicates increased randomness at the solid/solution interface during adsorption and that the absorbent had an affinity for the adsorbate [53]. The value of ΔH° is positive, indicating that the adsorption process was endothermic and more favourable at higher temperatures. According to Molina-Calderón, Basualto-Flores [39], if the value of ΔH° is between 20 and 80 kJ/mol, it indicates that adsorption is a combination of physisorption and chemisorption, typically associated with an ion-exchange mechanism. The ΔH° value was 41.26 kJ/mol, suggesting an ion-exchange mechanism for the adsorption of tetracycline onto ZNa-P1.

### 3.11. Adsorption Mechanism

**Figure 10:**
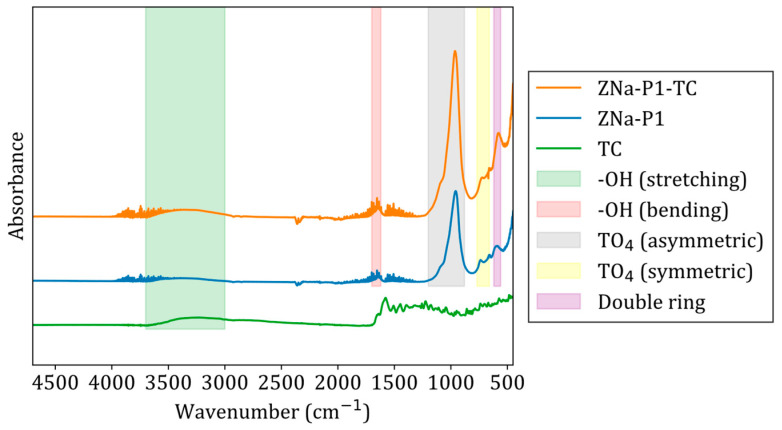
FTIR spectra of ZNa-P1 before and after adsorption of TC. Note that the FTIR spectra before adsorption are denoted as ZNa-P1 and the FTIR spectra after adsorption are denoted as ZNa-P1-TC.

Figure 10 gives the FTIR spectra of ZNa-P1 before and after adsorption of TC. The bands at 610–580 cm^−1^ are associated with the double ring vibration of zeolite Na-P1. The bands at 770–660 cm^−1^ correspond to the symmetric stretching vibration of TO_4_ (where T is Si or Al) [54]. The band at 1100–900 cm^−1^ corresponds to the asymmetric stretching vibration of TO_4_ [55]. The band at 1680–1620 cm^−1^ is attributed to the bending vibration of OH groups, while the broad band at 3700–3000 cm^−1^ is associated with the stretching vibration of OH groups [17].

After the adsorption of TC, shifts were noted in the peaks corresponding to the symmetric and asymmetric vibrations of TO_4_ at 770–660 cm^−1^ and 1100–900 cm^−1^, respectively. This indicated interactions between TC and ZNa-P1, conforming the adsorption of TC onto ZNa-P1. Additionally, a shift and an increase in the intensity of the stretching OH band, originally observed at 3700–3000 cm^−1^, was noted after adsorption. This suggested the presence of hydrogen bonding or other interactions between the hydroxyl groups in ZNa-P1 and the functional groups in TC [56]. The effect of pH revealed the presence of electrostatic attraction between TC and ZNa-P1, while adsorption thermodynamics indicated a potential ion-exchange mechanism.

Ultimately, the adsorption mechanism of tetracycline onto ZNa-P1 involved electrostatic attraction, hydrogen bonding, and ion exchange. Liu, Hou [57] used Zeolite A to adsorb TC and noted a similar adsorption mechanism, that being an ion-exchange process and electrostatic attraction.

## 4. Conclusions

This study evaluated the adsorption performance of three fly ash-derived adsorbents, AM-FA, BM-FA, and ZNa-P1, for tetracycline removal. The results demonstrated that hydrothermally synthesized ZNa-P1 was the most effective, achieving 90% tetracycline removal. Its superior performance was attributed to its high zeolite Na-P1 content (86%), which conferred a high cation exchange capacity (CEC = 6.37 meq/g) and a moderate surface area (35.75 m^2^/g). Notably, tetracycline adsorption was found to be more strongly correlated with CEC than with surface area.

Adsorption studies revealed that ZNa-P1 exhibited optimal performance at a dosage of 7.5 g/L and pH 5. The effect of pH in this study revealed that electrostatic attraction played a key role in TC adsorption. Kinetic modelling confirmed that the adsorption process followed the pseudo second order model (R^2^ = 0.98, SSE = 0.29). Adsorption isotherm studies indicated that tetracycline adsorption conformed to the Langmuir model, signifying monolayer adsorption on a homogeneous surface. Adsorption thermodynamics further established that the adsorption process was endothermic (positive ΔH°) and spontaneous (negative ΔG°), with increased disorder at the solid–liquid interface (positive ΔS°).

Overall, the adsorption of tetracycline onto ZNa-P1 was governed by electrostatic attraction, hydrogen bonding, and ion exchange. These findings highlight the potential of hydrothermally synthesized ZNa-P1 from fly ash as a promising, cost-effective adsorbent for tetracycline removal from aqueous solutions.

## Figures and Tables

**Table 1:** Summary of conditions for material synthesis.

Material	Reagents	Ratio	Procedure	Temperature	Duration	Post Treatment
AM-FA	30% HCl, Fly Ash	10 g FA:50 mL HCL	Mixed, placed in flask, heated in oven	100 °C	24 h	Vacuum Filtered, washed with DI water, dried at 60 °C for 24 h
BM-FA	3.5 M NaOH, Fly Ash	20 g FA:160 mL NaOH	Mixed, placed in autoclave, heated in oven	100 °C	24 h	Vacuum Filtered, washed with DI water (till pH < 10), dried at 60 °C for 24 h
ZNa-P1	3.5 M NaOH, Fly Ash	14 g FA: 100 mL NaOH	Step 1 (Ageing): Stirred in oil bath at 800 rpm	47 °C	48 h	-
	From Step 1	-	Step 2 (Hydrothermal): Added water, mixed, placed in autoclave, heated in oven	140 °C	48 h	Vacuum Filtered, washed with DI water (till pH < 10), dried at 60 °C for 24 h

**Table 2:** Summary of the adsorption experiments conditions and variables studied.

Adsorption Experiment	Adsorbent(s)	Conditions	Variable Studied
Preliminary Adsorption Test	FA, AM-FA, BM-FA, ZNa-P1	Co: 40 ppm, pH: 5, Dosage: 5 g/L, time: 2 h	Comparison of adsorbents
Effect of Dosage	ZNa-P1	Co: 40 ppm, pH: 5, Dosage: 1–12.5 g/L, time: 2 h	Dosage
Effect of pH	ZNa-P1	Co: 60 ppm, pH: 2–9, Dosage: 7.5 g/L, time: 2 h	pH
Adsorption Kinetics	ZNa-P1	Co: 60 ppm, pH: 5, Dosage: 7.5 g/L	Time
Adsorption Isotherms	ZNa-P1	Co: 70–120 ppm, pH: 5, Dosage: 7.5 g/L, time: 4 h	-
Adsorption Thermodynamics	ZNa-P1	T: 30 °C, 40 °C, 50 °C	Effect of temperature

**Table 3:** Chemical composition of FA, AM-FA, BM-FA, and ZNa-P1.

Oxides (wt%)	SiO_2_	Al_2_O_3_	MgO	Na_2_O	P_2_O_5_	Fe_2_O_3_	K_2_O	CaO	TiO_2_	SrO	LOI *
FA	49.88	31.01	1.79	0.36	0.86	3.52	0.84	4.73	1.51	0.53	4.46
AM-FA	57.68	27.62	0.59	0.25	0.47	1.26	0.81	0.97	1.62	0.29	7.95
BM-FA	38.17	28.64	1.70	9.62	0.44	3.25	0.49	4.30	1.39	0.50	11.01
ZNa-P1	40.62	27.76	1.5	14.65	0.24	2.12	0.18	4.42	1.44	0.12	6.33

* LOI—Lost on ignition.

**Table 4:** Specific surface area for FA, AM-FA, BM-FA, and ZNa-P1 using BET method.

Material	FA	AM-FA	BM-FA	ZNa-P1
Specific surface area (m^2^/g)	10.94	21.81	110.83	35.75

**Table 5:** Adsorption kinetic model parameters and statistical parameters for the adsorption of tetracycline.

Kinetic Model	Kinetic Parameters		Statistical Parameters		
Pseudo first order (PFO)	$q_{e}$ (mg/g)	$k_{1}$ (1/min)	Squared Error		R^2^
	7.83	0.58	1.17		0.93
Pseudo second order (PSO)	$q_{e}$ (mg/g)	$k_{2}$ (g/mg·min)	Squared Error		R^2^
	8.31	0.11	0.29		0.98
Langmuir kinetic	$k_{a}$ (L/g/min)	$k_{d}$ (mg/L/min)	$q_{max}$ (mg/g)	Squared Error	R^2^
	0.09	0.00	8.04	0.45	0.97

**Table 6:** Adsorption isotherm model parameters and statistical parameters for the adsorption of tetracycline onto ZNa-P1 at 30 °C, 40 °C, and 50 °C.

Isotherm Model	Temperature		
	30 °C	40 °C	50 °C
Langmuir isotherm model			
Isotherm Constants			
$q_{m}$ (mg/g)	46.34	48.79	49.14
$K_{L}$ (L/mg)	0.020	0.037	0.056
Statistical Parameters			
Squared Error	0.58	1.70	0.09
R^2^	0.973	0.967	0.998
Freundlich isotherm model			
Isotherm Constants			
$K_{f}$ (mg/g)	1.55	2.65	3.61
n	1.39	1.44	1.45
Statistical Parameters			
Squared Error	0.64	2.23	0.22
R^2^	0.971	0.957	0.995
Temkin isotherm model			
Isotherm Constants			
$B_{T}$ (J/mol)	0.22	0.36	0.56
$A_{T}$ (L/mg)	9.22	10.38	10.31
Statistical Parameters			
Squared Error	0.61	1.26	0.10
R^2^	0.972	0.976	0.998

**Table 7:** The maximum adsorption capacity of tetracycline for different adsorbents.

Absorbent	$q_{m}$ (mg/g)	pH	Temperature (°C)	Reference
Modified rubber waste (MRW)	76.33	3	25	[41]
Zeolite Na-P1 modified with β-cyclodextrin	38	-	-	[17]
Fly ash (FA)	5.26	7	25	[18]
Sophorolipid-base-modified fly ash (SFA)	142.97	-	25	[19]
Rice husk ash (RHA)	8.37	5	40	[45]
Activated carbon derived from Palm leave waste	132.94	5.86	25	[46]
Shrimp shell waste (SSW)	381.75	7	25	[47]
Na-montmorillonite	49.8	5.5	-	[48]
Polyvinyl chloride (PVC) Microplastics	21	7	-	[49]
Pumice stone	20.1	3	20	[50]
ZNa-P1	46.34	5	30	This study

**Table 8:** Thermodynamic parameters for tetracycline adsorption onto ZNa-P1.

Temperature (K)	$K_{eq}$	ΔG° (kJ/mol)	ΔH° (kJ/mol)	ΔS° (kJ/mol)	R^2^
303.15	9085.12	−22.97	41.26	0.21	0.993
313.15	16,490.54	−25.28			
323.15	24,983.65	−27.21			

## Data Availability

The raw data supporting the conclusions of this article will be made available by the authors upon request.
